# Automated lesion segmentation and quantification for prediction of paradoxical worsening in patients with tubercular serpiginous-like choroiditis

**DOI:** 10.1038/s41598-022-09338-y

**Published:** 2022-03-30

**Authors:** Gagan Kalra, Aniruddha Agarwal, Alessandro Marchese, Rupesh Agrawal, Reema Bansal, Vishali Gupta

**Affiliations:** 1 Cole Eye Institute, Cleveland Clinic, Cleveland, OH USA; 2 Eye Institute, Cleveland Clinic Abu Dhabi (CCAD), Abu Dhabi, UAE; 3 Department of Ophthalmology, San Raffaele Scientific Institute, IRCCS Ospedale San Raffaele, Vita-Salute San Raffaele University, Via Olgettina 60, 20132 Milan, Italy; 4 National Healthcare Group Eye Institute, Tan Tock Seng Hospital, Singapore, Singapore; 5 Singapore Eye Research Institute, Singapore, Singapore; 6 Moorfields Eye Hospital, NHS Foundation Trust, London, UK; 7 Lee Kong Chian School of Medicine, Nanyang Technological University, Singapore, Singapore; 8 Duke NUS Medical School, Singapore, Singapore; 9 Advanced Eye Center, Post Graduate Institute of Medical Education and Research (PGIMER), Chandigarh, India

**Keywords:** Retinal diseases, Uveal diseases

## Abstract

To develop and evaluate a fully automated pipeline that analyzes color fundus images in patients with tubercular serpiginous-like choroiditis (TB SLC) for prediction of paradoxical worsening (PW). In this retrospective study, patients with TB SLC with a follow-up of 9 months after initiation of anti-tubercular therapy were included. A fully automated custom-designed pipeline was developed which was initially tested using 12 baseline color fundus photographs for assessment of repeatability. After confirming reliability using Bland–Altman plots and intraclass correlation coefficient (ICC), the pipeline was deployed for all patients. The images were preprocessed to exclude the optic nerve from the fundus photo using a single-shot trainable WEKA segmentation algorithm. Two automatic thresholding algorithms were applied, and quantitative metrics were generated. These metrics were compared between PW + and PW- groups using non-parametric tests. A logistic regression model was used to predict probability of PW for assessing binary classification performance and receiver operator curves were generated to choose a sensitivity-optimized threshold. The study included 139 patients (139 eyes; 92 males and 47 females; mean age: 44.8 ± 11.3 years) with TB SLC. Pilot analysis of 12 images showed an excellent ICC for measuring the mean area, intensity, and integrated pixel intensity (all ICC > 0.89). The PW + group had significantly higher mean lesion area (*p* = 0.0152), mean pixel intensity (*p* = 0.0181), and integrated pixel intensity (*p* < 0.0001) compared to the PW- group. Using a sensitivity optimized threshold cut-off for mean pixel intensity, an area under the curve of 0.87 was achieved (sensitivity: 96.80% and specificity: 72.09%). Automated calculation of lesion metrics such as mean pixel intensity and segmented area in TB SLC is a novel approach with good repeatability in predicting PW during the follow-up.

## Introduction

With 10 million individuals developing the disease in 2019 alone, tuberculosis is still a globally prevalent infectious disease^1^. Tubercular serpiginous-like choroiditis (TB SLC) is an inflammatory disease, thought to be an immune response to *Mycobacterium tuberculosis,* affecting the choriocapillaris, choroid and the retinal pigment epithelium in a recurrent course in patients with systemic or latent tuberculosis^2–6^.

Two distinct phenotypes of TB SLC include multifocal, and placoid based on the lesion morphology. These phenotypes have different clinical course, for instance in the multifocal type, distinct lesions form with an active edge progressing to coalesce whereas in the placoid phenotype, a single placoid lesion enlarges in a centrifugal pattern. Both the phenotypes, however, show an active yellow edge with a central healing. Further, paradoxical worsening of TB SLC lesions may be seen on initiation of anti-tubercular therapy (ATT). Paradoxical worsening is a major concern in these cases as it may cause severe visual loss and requires high-dose corticosteroids along with adjunct intravitreal injections or immunosuppressive therapy to prevent permanent damage^6–8^.

There is sparse literature available for prediction of outcomes upon initiation of therapy based on baseline imaging and clinical characteristics of choroiditis lesions. In our previous work, we determined the role of anatomic characteristics of the TB SLC active lesion at baseline to qualitatively predict response to therapy and progression^9^. In our current study, we developed and implemented a fully automated pipeline using baseline-visit color fundus images in patients with TB SLC to segment lesions and generate quantitative biomarkers that can predict paradoxical worsening in response to ATT.

## Materials and methods

This study adheres to the declaration of Tenets of Helsinki and was performed after receiving approval from the Institutional Ethics Committee (IEC) of Post Graduate Institute of Medical Education and Research (PGIMER), Chandigarh, India. Written informed consent was obtained from all the study subjects included.

In a retrospective study design, clinical data and images from subjects with newly diagnosed TB SLC were included in the analysis. The diagnosis was established based on clinical history, examination, imaging and laboratory investigations. TB SLC patients with a minimum of 2 follow-up visits at 1 month and 9 months after initiation of ATT were included in the analysis to assess the effect of therapy and monitor disease activity. Patients with shorter total period of follow-up were excluded. Eyes with media opacity (due to small pupil, cataract or other reasons) and poor fixation precluding adequate imaging quality were excluded. All patients underwent complete ocular examinations including best-corrected Snellen’s visual acuity (BCVA), slit-lamp biomicroscopy and indirect ophthalmoscopy as well as ocular imaging including color fundus photography (Visupac FF450, Carl Zeiss Meditec, Jena, Germany).

Paradoxical worsening was defined as worsening of disease upon initiation of ATT within first 3 months characterized by development of new choroiditis lesions or worsening of previous lesions with increased size/number. Based on presence ( +) or absence (-) of paradoxical worsening, a ground truth binary classification was established by expert graders (VG and AA). Thus, the patients were categorized as either “PW + ” or “PW-”. All the images were anonymized, coded, and randomly assorted (both initial and follow-up visits so that it was difficult to ascertain the timeline by the image analyst). Image analysts and statistician (GK) were blinded to the ground truth classification.

### Pilot analysis

**Figure 1:**
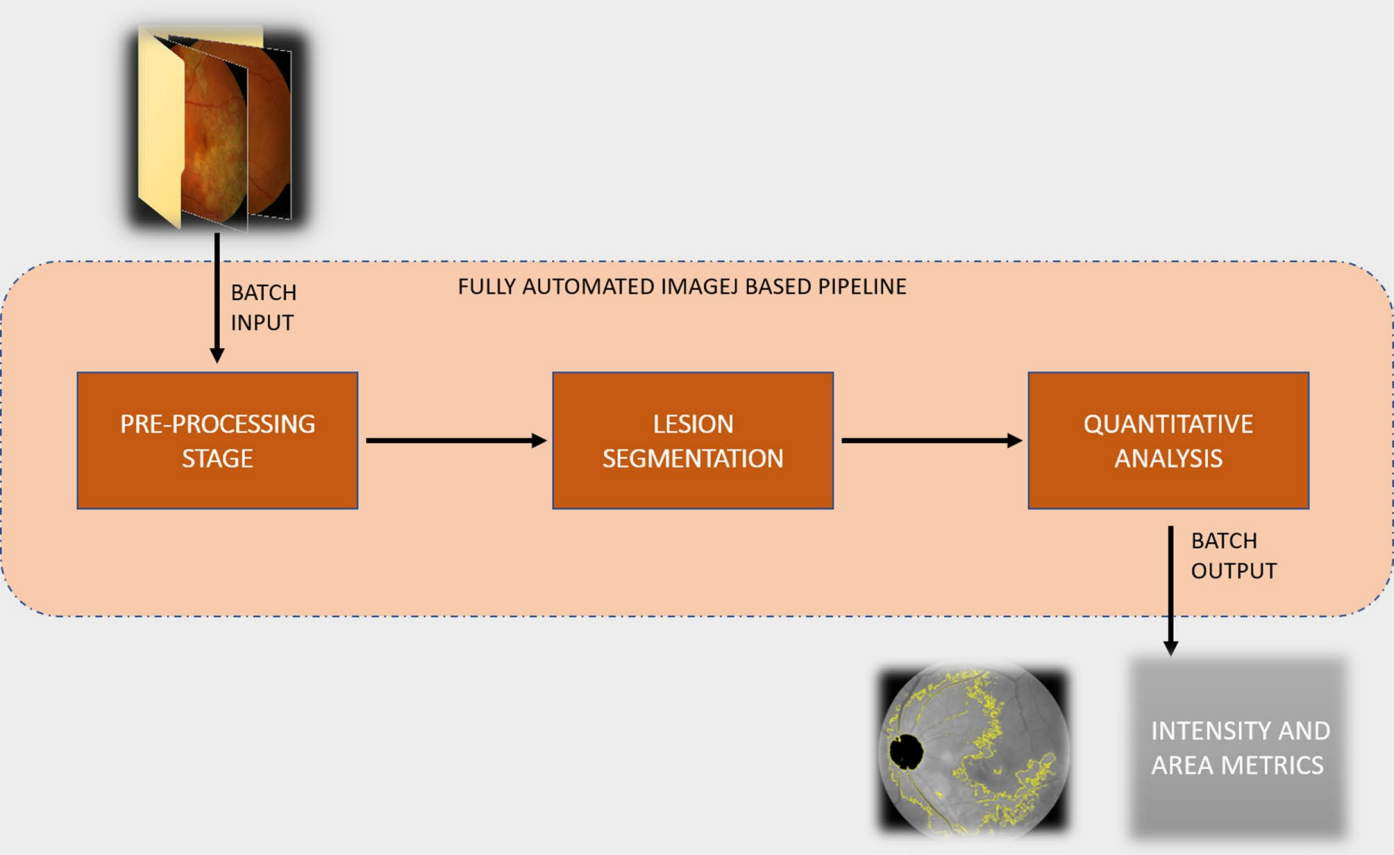
Schematic describing our workflow for the classification pipeline.

A fully automated custom-designed ImageJ (ImageJ, National Institutes of Health, Bethesda, USA)-based pipeline was developed that performed image pre-processing, lesion segmentation and quantitative analysis (Fig. 1) using color fundus photography at baseline visit for 12 images (6 patients) as a pilot. Two distinct images of the same eye captured at the baseline visit, were analyzed using the pipeline for assessment of repeatability. Bland–Altman plots were generated and intraclass correlation coefficient (ICC) was calculated to measure agreement. An ICC below 0.5 was considered poor, 0.5–0.6 was considered fair, > 0.6 was considered good and > 0.8 was considered to have excellent agreement between scans. After confirming adequate repeatability agreement, the pipeline was deployed for all patients included in this study.

### Imagej based pipeline

**Figure 2:**
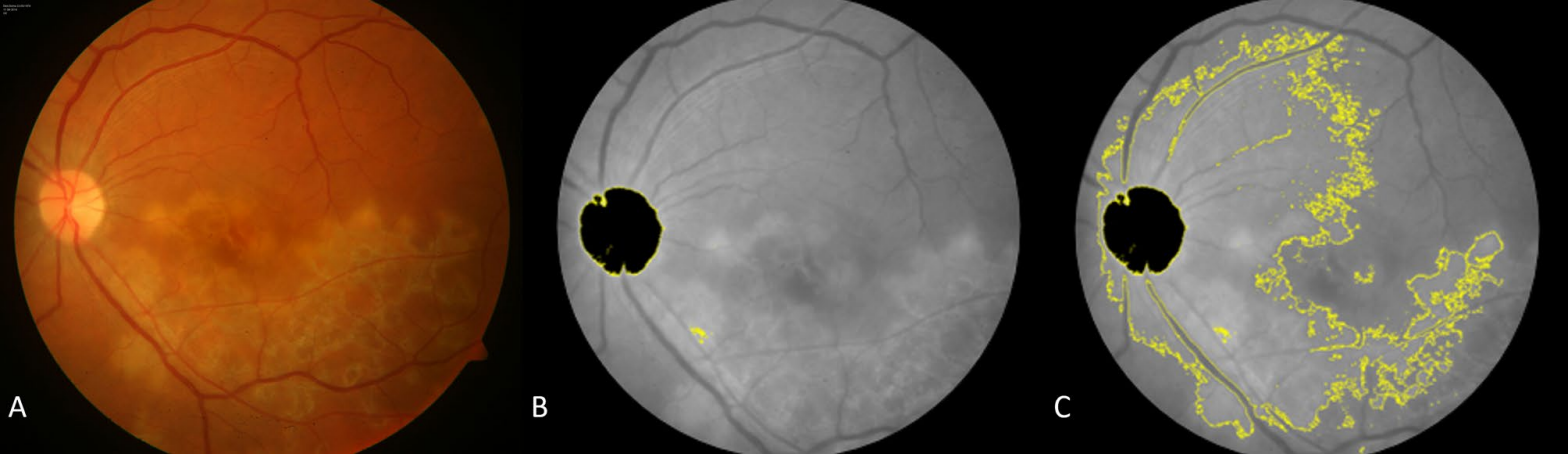
(**A**) Color fundus photograph of a representative case with TBSLC lesion; (**B**) Pre-processed image; (**C**) Lesion area selection obtained after image segmentation step.

In the pre-processing stage, all images were imported and converted to 8-bit for further analysis. Image contrast was enhanced using the built-in ImageJ tool—“Enhance contrast” under “Process” menu. The value of 0.3% was used for contrast enhancement and pixel intensities for all images were normalized on a 256-step scale between 0 and 255 to minimize disparities due to capture exposure differences. The image normalization, performed by redistributing the pixel intensity values across a uniform scale, preserves the relationships between pixel intensity between neighboring pixels while still offsetting any differences that may have occurred during the process of image capture (unlike histogram equalization). Further, a peripheral rim of the image equating to about 5 – 8 degrees FOV (field-of-View) was excluded from analysis to account for peripheral artifacts. Next, a single shot WEKA segmentation algorithm was deployed to identify, segment and subsequently exclude the optic nerve head from further analysis (Fig. 2). This algorithm is based on random forest architecture and has been previously described in the literature^10,11^. The image thus obtained would be referred to as pre-processed images in this manuscript.

In the lesion segmentation stage, pre-processed images were subject to sequential multi-level thresholding involving an initial less specific automatic thresholding algorithm, namely Shanbhag’s threshold, followed by a more specific automatic thresholding algorithm, namely Li’s threshold. The resulting selection delineated the lesion area (Fig. 2C).

In the quantitative analysis stage, the pipeline generated quantitative metrics by running “Measure” in ImageJ in the area of the selection made at the previous step. Metrics obtained included mean pixel intensity, integrated pixel intensity, minimum pixel intensity, maximum pixel intensity and segmented lesion area, which were subsequently compared between PW + and PW- groups. Mean pixel intensity indicates mean of pixel intensities in the selected region of interest while the maximum and minimum pixel intensity indicate the maxima and minima of pixel intensity in the selected region of interest. Lesion area indicates the area of selected region of interest (in pixel squared) and integrated pixel intensity is the product of mean pixel intensity and lesion area.

### Statistical analysis

Statistical analyses were deployed in R software (v4.0.3, John Chambers and colleagues, Bell Laboratories). Box-and-whiskers plots were utilized to visualize the data. Parametric and nonparametric tests were utilized to compare means in the generated quantitative metrics between groups PW + and PW-. A logistic regression model was used to assess binary classification performance and receiver operator curves (ROC) were generated to choose sensitivity-optimized threshold. Statistical significance was inferred for a p-value of less than 0.05.

## Results

In this study, 139 patients (139 eyes; 92 males and 47 females) with TB SLC were included. All the subjects were Asian Indian in ethnicity. The mean age of all the subjects was 44.8 ± 11.3 years. The demographic characteristics have been described in Table 1.

**Table 1:** Demographic and clinical characteristics of patients with tubercular serpiginous-like choroiditis in PW + and PW- groups.

Variable		PW + group	PW- Group	*P* value
Number of patients (n)		44	95	–
Age (years ± stdev)		42.94 ± 7.84	46.07 ± 6.23	0.66
**Gender**				
Male (n)		27	62	0.83
Female (n)		14	33	
Duration of symptoms (weeks)		3.81 ± 2.29	4.07 ± 3.16	0.52
Baseline BCVA (LogMAR)*		0.92 ± 0.31	0.87 ± 0.39	0.76

*: Best Corrected Visual Acuity.

## Repeatability and intraclass correlation

**Figure 3:**
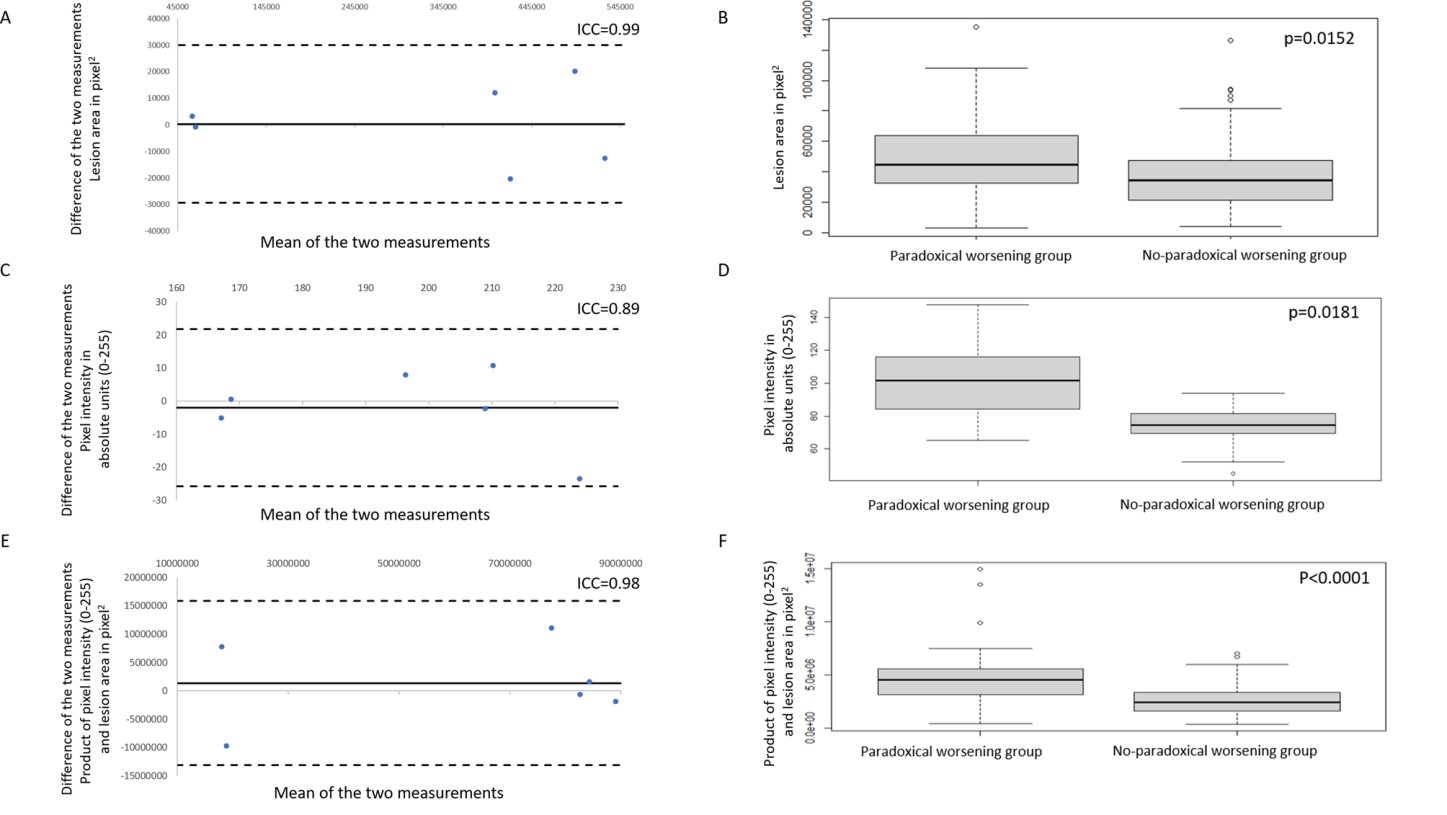
(**A**), (**C**), and (**E**) show Bland–Altman plots for the pilot analysis comparing measurements of lesion area, mean pixel intensity, and product of lesion area and mean pixel intensity respectively, from two images from each of the 6 patients from the same visit. (**B**), (**D**), and (**F**) show box and whisker plots for lesion area, mean pixel intensity, and product of lesion area and mean pixel intensity respectively.

The analysis of 12 pilot images from 6 patients, 2 image instances for each eye at baseline visit, showed that the pipeline had an excellent ICC of 0.99 for measuring the area of the lesion, 0.89 for the mean intensity and 0.98 for integrated pixel intensity (Table 2). Bland Altman plots for these measurements showed excellent agreement are represented in Fig. 3A, C and E respectively.

**Table 2:** Repeatability analysis from 12 images from 6 patients (6 eyes) from the fully automated ImageJ based analysis.

Metric	Mean pixel intensity*	Area of lesion**	Integrated pixel intensity^
ICC†	0.89	0.99	0.98

*: Indicates mean of pixel intensities in the selected region of interest.

**: Indicates the area of selected region of interest (in pixel squared).

^: Integrated pixel intensity: Indicates the product of Mean Pixel Intensity and Area of Lesion.

^†^ICC: Intraclass Correlation Coefficient to assess repeatability.

### Quantitative metrics

**Figure 4:**
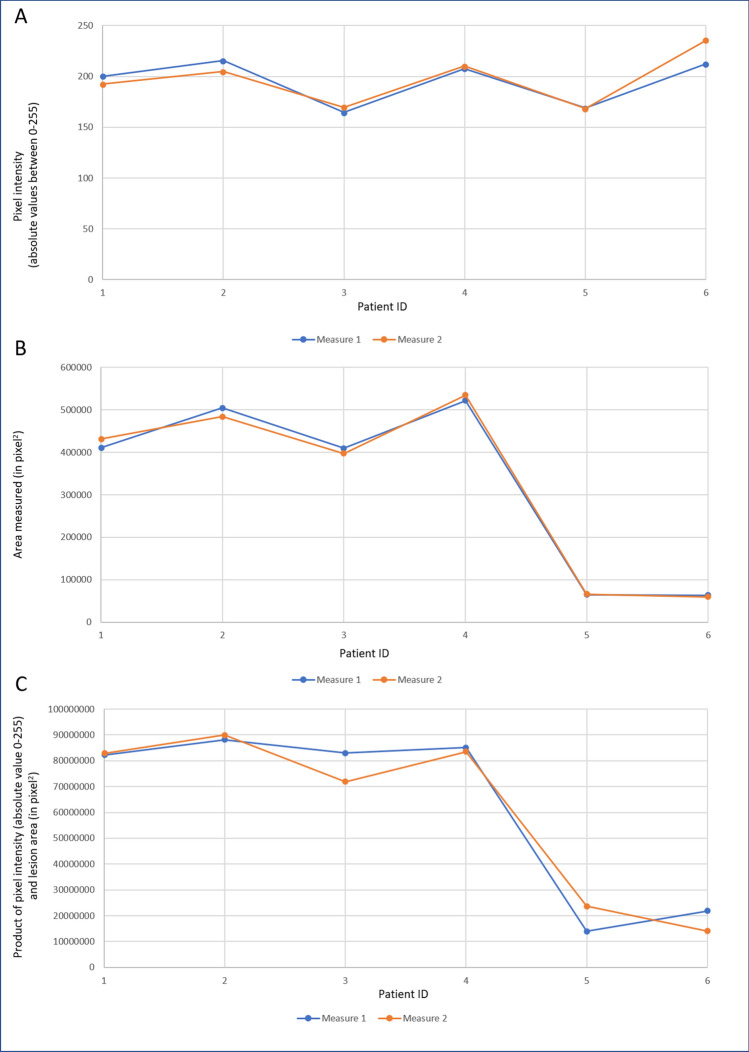
Scatter plots with linear relation lines for (**A**) mean pixel intensity; (**B**); lesion area, and (**C**) product of mean pixel intensity and lesion area.

Mean lesion area between the groups showed significantly higher measurements (*p* = 0.0152) for PW + group compared to PW- group. Box-and-whiskers plot for lesion area is depicted in Fig. 3B. Statistically significant difference was also noted (*p* < 0.0001) for minimum and maximum pixel intensity between groups. The difference in the mean pixel intensity between the two groups was significant (*p* = 0.0181) with the PW + group having the higher mean pixel intensity in the selected ROI (Fig. 3D). Integrated pixel intensity between the groups (Fig. 3F) had statistically significant difference (*p* < 0.0001) with higher mean values in the PW + group. These results are summarized in Table 3. Scatter plots with linear relation lines are shown in Fig. 4. Correlation coefficient for lesion area with the intensity was found to be -0.05 with a p-value of 0.53 not meeting statistical significance.

**Table 3:** Comparison of colour fundus photography quantitative biomarkers between PW + and PW- groups.

Imaging biomarker	Paradoxical Worsening (PW +) group [Mean (95% CI)]	Non-paradoxical Worsening (PW-) group [Mean (95% CI)]	*P*-value for comparison between groups
Mean Pixel Intensity*	102.25 (94.75–110.14)	74.44 (67.05–71.83)	0.0181
Minimum Pixel Intensity**	91.02 (85.56–96.49)	71.68 (69.61–73.76)	< 0.0001
Maximum Pixel Intensity^	155.30 (147.30–163.30)	78.76 (76.98–80.53)	< 0.0001
Area of Lesion†	49,265 (40,890–57,639)	37,374 (32,668–42,080)	0.0152
Integrated Pixel Intensity‡	4,843,904 (3,972,554–5,715,255	2,687,416 (2,375,891–2,998,941)	< 0.0001

*: Indicates mean of pixel intensities in the selected region of interest.

**: Indicates the minima of pixel intensities in the selected region of interest.

^: Indicates the maxima of the pixel intensities in the selected region of interest.

^†^: Indicates the area of selected region of interest (in pixel squared).

^‡^: Indicates the product of Mean Pixel Intensity and Area of Lesion.

**Figure 5:**
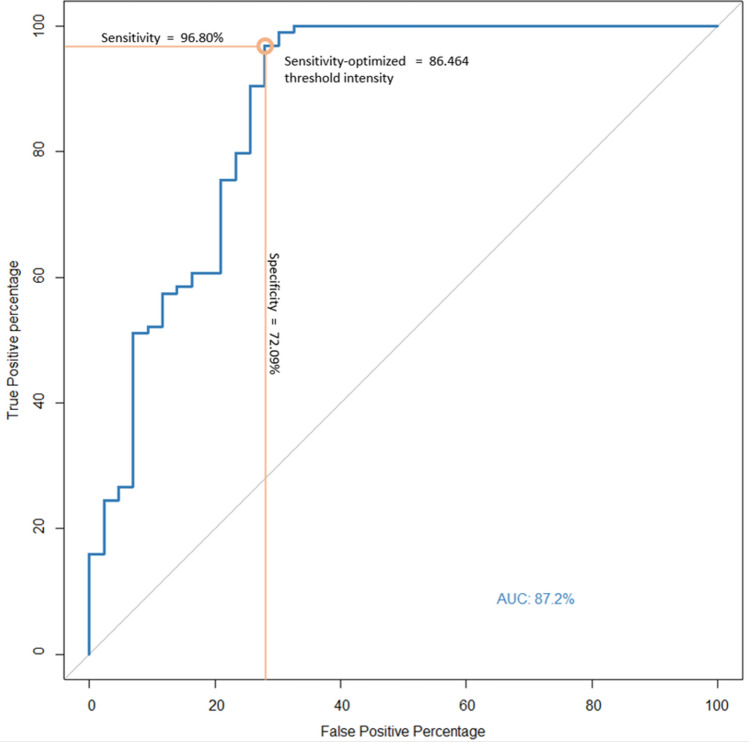
Receiver operator curve (ROC) and area under curve (AUC) for identifying PW + and PW- patients using mean pixel intensity measured using our algorithm. True positivity percentage or sensitivity is represented across the y-axis and false positivity percentage or (1-specificity) is represented across the x-axis.

ROC curve (Fig. 5) was generated using a univariate logistic regression model based on mean pixel intensity for binary classification into PW + and PW- groups. An area under the curve (AUC) of 0.87 was achieved with a sensitivity of 96.80% and specificity of 72.09% using a sensitivity optimized threshold cut-off value for mean pixel intensity of 86.464.

## Discussion

The significant visual morbidity associated with TB SLC led to a cause of concern globally among uveitis specialists who together formed the Collaborative Ocular Tuberculosis Study (COTS) group^12–15^. The COTS identified the importance of the serpiginous phenotype, which was agreed to be the most distinctive manifestation of ocular TB universally by all the uveitis specialists.^16,17^ Presenting as a yellow-white fuzzy outer retinal lesion with active serpentine edges and healing center, the disease classically manifests in individuals from Asian Indian and Middle Eastern ethnicities and positive immunological tests for TB^3,5,17,18^.

One of the major challenges in the management of TB SLC is the development of paradoxical worsening of the disease, which typically occurs within the first 3 months of initiating ATT^7,19–21^. Paradoxical worsening can be differentiated from continuous progression of the disease since it has an initial phase of improvement and reduction of the inflammatory lesions. However, paradoxical worsening can have deleterious effect on central vision because new lesions can appear in the central macula or in the peripapillary retina, resulting in permanent functional compromise. Therefore, an important aspect in the management of TB SLC is the rapid identification and quantification of paradoxical worsening and its treatment with high-dose systemic or local corticosteroids/immunosuppressants^7,19–21^. Paradoxical worsening can be predicted by various imaging biomarkers including optical coherence tomography angiography (OCTA) and inflammatory markers from the vitreous such as interleukins and tumor necrosis factor.^8,22^ Studies have shown that early detection/prediction of paradoxical worsening can prevent permanent choriocapillaris/photoreceptor atrophy and help in superior visual outcomes^8,22^.

Our previous study analyzed patients with TB SLC using a self-designed grading scale based on lesion opacity and yellowness on color fundus photographs^9^. The study showed that lesions with high opacity grades, showing intense yellow opacification of the active borders, were associated with statistically higher risks of suboptimal response to treatment, lesion progression, and paradoxical worsening during the course of follow-up. This study showed that grading of the lesions at the initial presentation can be valuable in determining the course of the disease as well as functional and anatomical outcomes^9^.

In the current study, an automated pipeline was designed to delineate serpiginous lesions due to TB in color fundus photographs. As the first step, two image instances of the same eye at baseline were analysed using the pipeline for each of the 6 patients. Various metrics chosen for the analyses included pixel intensity (minimum, maximum and mean), integrated pixel intensity, and segmented lesion area. The ICC obtained from the automated analysis of the images was excellent, and the same pipeline was subsequently applied to the entire batch of fundus photographs. The derived quantitative metrics revealed that in the paradoxical worsening group, the baseline images had higher segmented lesion area and mean pixel intensity values. These results are consistent with our previous manual grading^9^, with an ROC curve showing high sensitivity of approximately 97% for detection of paradoxical worsening.

There are several advantages of this automated approach of calculating choroiditis lesion metrics: first, such an approach of identification of choroiditis lesion boundaries using freely available third-party software such as ImageJ has not been reported in the literature. This fully automated pipeline can be deployed for rapid assessment of hundreds of images within minutes. Moreover, such an analysis can form the basis of developing deep learning pipelines to predict which eyes are at the highest risk of developing paradoxical worsening. The second advantage of this approach is that the clinician can be forewarned regarding the risk of development of paradoxical worsening once ATT is initiated. The clinician can, therefore, be very cautious at the time of reduction of oral corticosteroid therapy, especially during the crucial period of 3 to 8 weeks after initiation of ATT. The third advantage of this technique is that it is completely objective and does not require a trained uveitis specialist to grade the images. Owing to this, the image analysis can also be done remotely using telemedicine for guiding the treating ophthalmologist from a distant location.

The lesion intensity and area as measured using the ImageJ pipeline seem to be quasi-markers of immunogenicity since they represent the “activity” and level of inflammation. As shown in our previous study^9^, and the study by Holland et al. in eyes with cytomegalovirus retinitis^23^, assessment of lesion “opacity” is relevant because it directly correlates with systemic infection, lesion size, and high-risk factors such as bilaterality. Therefore, lesion pixel intensity can serve as a novel biomarker of disease activity in TB SLC.

Our study has a number of limitations, including a retrospective study design. A retrospective study design suffers from weaknesses due to possible flaws in record-keeping and documentation. It is likely that selection bias can also occur in this study design since several patients may have been excluded due to the pre-defined inclusion criteria. Those patients with more severe disease may have had a better follow-up record and could have undergone complete imaging evaluation at every visit. We took only those subjects with posterior disease and analyzed the lesions in the central 45-degrees only. We had to crop the peripheral 5–8 degrees of the fundus photograph to avoid miscalculation of the choroiditis lesions due to peripheral artefacts. This may have led to inaccuracies in estimating the actual lesion area. In addition, patients with peripheral disease may have been excluded from the analysis, leading to a bias in the results^24,25^. In this study, we used images from a single fundus camera, and it is possible that quantitative results may vary based on the device used. However, our method first converts the color fundus images into an 8-bit black and white image before then subjecting it to a normalization algorithm in ImageJ. This step is critical as it considers the different brightness intensities within the individual image and redistributes it across a known scale to achieve normalized images. Although the current study does not test this, we believe that since our method relies on the custom brightness values within each individual image, it should help to offset differences, if any exist, between images coming from different devices. Since our analysis is based on a thresholding method and it is not specifically designed to detect choroiditis lesions using artificial intelligence, there are chances that the thresholding algorithm may fail at several locations within a single image frame. It is likely that the area of choroiditis may have been inaccurately left out and normal retinal areas included after the two-step thresholding. A comparison with manual delineation of the lesion may help validate the ImageJ analysis. Further, a prospective study can help in determining the predictability of this pipeline in truly identifying paradoxical worsening. Other imaging modalities such as fundus autofluorescence, fluorescein angiography, and OCTA are also very useful in detecting paradoxical worsening. However, in this study, we did not explore other imaging modalities and cannot comment on their utility in improving our classification algorithm. In a screening setting, our algorithm can be deployed using the widely available fundus photography when more specialized imaging may not be available.

In summary, automated calculation of choroiditis lesion metrics is a novel approach which showed excellent repeatability in the analysis of TB SLC lesions. Lesions which developed paradoxical worsening during the follow-up had higher baseline mean lesion pixel intensity and segmented area. This pipeline also showed a high sensitivity in detecting paradoxical worsening in the follow-up with a numerical value of pixel intensity at baseline. In the future, this image analysis technique shows promise and can be applied in other conditions as well.
